# SARS-CoV-2 accessory protein 7b forms homotetramers in detergent

**DOI:** 10.1186/s12985-022-01920-0

**Published:** 2022-11-21

**Authors:** Wahyu Surya, Maria Queralt-Martin, Yuguang Mu, Vicente M. Aguilella, Jaume Torres

**Affiliations:** 1 School of Biological Sciences, Nanyang Technological University, 60 Nanyang Drive, Singapore, 637551 Singapore; 2 Laboratory of Molecular Biophysics, Department of Physics, Universitat Jaume I, 12080 Castelló, Spain

**Keywords:** Accessory protein 7b, SARS-CoV-2, COVID-19, Analytical ultracentrifugation, Channel activity, Coronavirus

## Abstract

A global pandemic is underway caused by the severe acute respiratory syndrome coronavirus 2 (SARS-CoV-2). The SARS-CoV-2 genome, like its predecessor SARS-CoV, contains open reading frames that encode accessory proteins involved in virus-host interactions active during infection and which likely contribute to pathogenesis. One of these accessory proteins is 7b, with only 44 (SARS-CoV) and 43 (SARS-CoV-2) residues. It has one predicted transmembrane domain fully conserved, which suggests a functional role, whereas most variability is contained in the predicted cytoplasmic C-terminus. In SARS-CoV, 7b protein is expressed in infected cells, and the transmembrane domain was necessary and sufficient for Golgi localization. Also, anti-p7b antibodies have been found in the sera of SARS-CoV convalescent patients. In the present study, we have investigated the hypothesis that SARS-2 7b protein forms oligomers with ion channel activity. We show that in both SARS viruses 7b is almost completely α-helical and has a single transmembrane domain. In SDS, 7b forms various oligomers, from monomers to tetramers, but only monomers when exposed to reductants. Combination of SDS gel electrophoresis and analytical ultracentrifugation (AUC) in both equilibrium and velocity modes suggests a dimer-tetramer equilibrium, but a monomer–dimer–tetramer equilibrium in the presence of reductant. This data suggests that although disulfide-linked dimers may be present, they are not essential to form tetramers. Inclusion of pentamers or higher oligomers in the SARS-2 7b model were detrimental to fit quality. Preliminary models of this association was generated with AlphaFold2, and two alternative models were exposed to a molecular dynamics simulation in presence of a model lipid membrane. However, neither of the two models provided any evident pathway for ions. To confirm this, SARS-2 p7b was studied using Planar Bilayer Electrophysiology. Addition of p7b to model membranes produced occasional membrane permeabilization, but this was not consistent with bona fide ion channels made of a tetrameric assembly of α-helices.

**Supplementary Information:**

The online version contains supplementary material available at 10.1186/s12985-022-01920-0.

## Introduction

Coronaviruses (CoV) are vertebrate pathogens which cause human respiratory diseases that typically affect the respiratory tract and gut. They have been kown to cause common cold symptoms in humans, and a variety of lethal diseases in birds and mammals [1]. However, in 2003, the virus responsible for the severe acute respiratory syndrome (SARS-CoV) [2], referred hereafter as SARS, produced a near pandemic with 8,098 infected and 774 deaths, i.e., a 10% mortality rate [3]. Currently, a global pandemic of Coronavirus disease 19, i.e., COVID-19, (https://www.who.int/health-topics/coronavirus) caused by SARS-CoV-2, hereafter SARS-2 [4], is underway at the time of writing this manuscript, infecting 410 million people and causing more than six million deaths [5]. It is important to urgently explore all possible pharmaceutically accessible therapeutic targets in SARS-2 proteins and host interactions [6]. CoVs belong to the family *Coronaviridae*, subfamily *Coronavirinae,* and are distributed into four genera [7]. In CoVs genomes, the first two thirds encode non-structural genes; open reading frames ORF1a and ORF1b produce polyproteins pp1a and pp1ab, which are processed into 16 nonstructural proteins (nsp1 to 16). The last third of the genome hosts the ORFs for structural proteins: spike (S), envelope (E), membrane (M) and nucleoprotein (N), and also other so-called ‘accessory’ proteins, which vary in number and sequence even among CoVs belonging to the same lineage [8–10].

Specific for SARS-CoVs are eight ORFs that encode accessory proteins, namely ORFs 3a, 3b, 6, 7a, 7b, 8a, 8b and 9b [11, 12]. Although these proteins have been considered not essential for viral replication in vitro [13–15], several of these have been found to be involved in virus-host interactions during infection in vivo [13, 16]. Accessory proteins may confer biological advantages to the virus in the natural host, and contribute to pathogenesis [11]. In SARS, protein 7b (p7b thereafter) was predicted to be translated by leaky scanning from a second ORF present in SARS-CoV sgRNA7 [17], and expression was confirmed in infected Vero cells [18]. Although no experiments have been performed to detect the expression of p7b in tissue samples from SARS patients, the presence of anti-p7b antibodies in SARS convalescent patient sera indicates that the p7b is likely expressed in vivo [19], and has been reported to be present in purified virions [18]. In SARS, p7b is 44 amino acids long and has a highly hydrophobic polypeptide predicted to span the membrane, with a luminal N-terminus and a cytoplasmic C-terminus [18]. The localization of p7b is similar to that of p7a, and found throughout the Golgi compartment in both SARS-infected cells and in cells transfected with 7b cDNA [18]. It is incorporated to the SARS virion, but it is not detected on the cell surface of the transfected cells [18]. The transmembrane domain of p7b, specifically residues 21–23 and 27–30, was found to be both necessary and sufficient for its Golgi localization [20]. SARS ORF7b was not found to be essential for replication in vitro or in vivo [15, 18, 21]. However, a prototype virus (strain Frankfurt-I) isolated during the 2003 SARS outbreak [22] had a 45-nt deletion in the transmembrane domain of ORF7b and a replicative advantage in some cells [23], suggesting an attenuating role for p7b. Also, studies using siRNA specific for SARS-CoV sgRNA7 showed silencing of the expression of 7a, 7b, 8a and 8b [24], indicating that p7a/p7b (and p8a/p8b) may play certain roles during the SARS replication cycle. It has been shown that p7b can induce apoptosis in infected cells [25], but the significance of this in the viral life cycle is not clear [26].

**Fig. 1:**
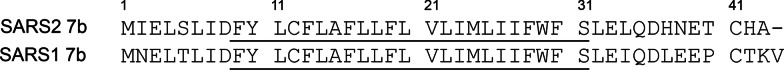
Sequences of SARS2 and SARS p7b, where the predicted transmembrane domain (TMD) is underlined

The current COVID-19 global pandemic is caused by SARS-2 which, like its SARS predecessor, also encodes accessory proteins. The sequence of p7b in SARS-2 is one residue shorter (43 residues) than the one in SARS, and their predicted transmembrane domain is fully conserved (Fig. 1), suggesting a functional role. Overall, sequence identity is 88%, where > 90% of the variability is contained in the predicted cytoplasmic C-terminus. Recently, SARS-2 p7b has been shown to mediate apoptosis in cells mediated by tumor necrosis factor-α (TNF-α) [27]. However, with the exception of one study showing SARS-2 7b forms oligomers in SDS gels, and a proposal of a hypothetic pentameric model similar to phospholamban, no further experimental characterization of p7b is available in the literature [28].

In the present study, we have investigated the hypothesis that SARS-2 p7b forms oligomers with ion channel activity. Oligomeric size was determined for the first time using analytical ultracentrifugation (AUC) in the sedimentation equilibrium (SE) and sedimentation velocity (SV) modes, whereas possible channel activity was tested using planar lipid bilayers. Finally, we suggest a preliminary model for the interaction of the 7b monomers obtained by a molecular dynamics simulation performed in presence of a lipid membrane.

## Materials and methods

### Peptide purification and reconstitution

The 43-residue long SARS-2 p7b was obtained as a crude peptide, synthesized with amidated C-terminus and free N-terminus (Genscript, USA). SARS p7b was synthesized in-house with amidated C-terminus and free N-terminus using microwave-assisted solid-phase fluorenylmethyloxycarbonyl (FMOC) chemistry using an Odyssey Microwave peptide synthesizer (CEM corporation, US). The protein was cleaved from the resin with trifluoroacetic acid (TFA) and lyophilized. The peptides were dissolved in TFA (10 μL) followed by dilution with acetonitrile to a final concentration of 5 mg/mL. The solution was injected into a C4-300 Å reverse-phase high-performance liquid chromatography (RP-HPLC) column (Phenomenex, Cheshire, UK) connected to a HPLC system (Shimadzu, Japan). The solvents used were solvent A: water with 0.1% TFA (v/v), and solvent B: isopropanol/acetonitrile (4:1 v/v) with 0.1% TFA (v/v). The peptide was eluted with a linear gradient from 30 to 75% of solvent B. Pooled fractions were lyophilized and the purity of the samples was checked by MALDI-TOF MS. The transmembrane domain (p7b-TM) was synthesized and purified in the same way.

### Reconstitution in membranes

Reconstitution of p7b in lipid membranes was performed first by mixing the lyophilized protein in TFE with LPR_m_ (molar lipid-to-protein ratio) of 20 for DMPC lipid or ‘ERGIC lipid mixture’ (POPC: POPE: bovine PI: POPS: Cholesterol, in a molar ratio 45:20:13:7:15) in chloroform. Lipids were purchased from Avanti Polar Lipids (Alabaster, US). The mixture was dried under a N_2_ stream and incubated in vacuum overnight before resuspension in water by vortexing and freeze-thawing. Reconstitution of 7b-TM was achieved by mixing ethanol-dissolved lipid and peptide. The solvent was then evaporated with N_2_ gas and the sample was rehydrated in water.

### Infrared spectroscopy

FTIR spectra were recorded on a Nicolet Nexus 560 spectrometer (Madison, USA) purged with N_2_ and equipped with a MCT/A detector cooled with liquid nitrogen. Attenuated total reflection (ATR) spectra were measured with a 25-reflections ATR accessory from Graseby Specac (Kent, UK) and a wire grid polarizer (0.25 mM, Graseby Specac). Approximately 100 μL of sample in water at 20:1 LPR molar ratio were applied onto a trapezoidal (50 × 2 × 20 mm) Ge internal reflection element (IRE). A dry, or D_2_O saturated, N_2_ stream flowing through the ATR compartment was used to remove bulk water or to achieve D_2_O exchange, respectively. A total of 200 interferograms collected at a resolution of 4 cm^−1^ were averaged for every sample and processed with one-point zero filling and Happ-Genzel apodisation. The % of amino acids embedded in the membrane was obtained from an amide hydrogen–deuterium exchange experiment, where the lipid/protein film was subjected to a flow of D_2_O saturated nitrogen for 30 min. The area of the amide II (N–H bending, centered at ~ 1550 cm^−1^) and amide I (C=O stretching, centered at ~ 1655 cm^−1^) bands was obtained by peak integration from 1510 to 1590 cm^−1^ and 1600 to 1700 cm^−1^ The fraction of non-exchanged residues was determined as described previously [29].

### Gel electrophoresis

The peptide samples were solubilized in NuPAGE sample buffer, with or without reductant, 5 mM Tris(2-carboxyethyl)-phosphine (TCEP) or dithiothreitol (DTT), and run on a 13.5% Bis–Tris gel following the NuPAGE protocol (Invitrogen, Thermo Fisher Scientific). The gel was stained with Coomassie blue G-250.

### Analytical ultracentrifugation (AUC)

AUC sedimentation velocity (AUC-SV) experiments were performed using a Beckman ProteomeLab XL-I analytical ultracentrifuge with a rotor An-50Ti. p7b samples were reconstituted in 5 mM myristyl sulfobetaine (C14SB, Sigma), 50 mM Tris pH 7.3 and 100 mM NaCl, with or without addition of 2 mM TCEP, and in presence of 29.4% (v/v) D_2_O to eliminate detergent buoyancy. The samples were centrifuged at 50,000 rpm in epon 2-sector centrepiece AUC cells with quartz windows. Absorbance profile at 280 nm was collected every 10 min for 15 h. Sedimentation profiles were analysed in SEDFIT using the c(s) model [30] and plotted with GUSSI [31]. The S-values corresponding to monomer, dimer, or tetramer of p7b in C14SB micelles were predicted considering the properties of detergent, protein and buffer composition. The molecular weight (MW), aggregation number and specific volume of C14SB detergent was 363.6 Da, 83–130 (www.anatrace.com) and 0.965–0.978 mL/g (based on our density matching data), respectively. Using the sequence of SARS2-7b, the MW is 5180 Da and the specific volume is 0.7702 mL/g (calculated using Sednterp software). The density and viscosity of the buffer (50 mM Tris, 100 mM NaCl and 29.4% D_2_O) was ρ = 1.0353 g/mL and η = 1.0997 cP (calculated using Sednterp software), respectively. Assuming the lowest estimate for the number of detergent molecules bound and lowest estimate of C14SB specific volume (ν_D_) and lowest specific volume of the micelle (ν_c_), the weight of the micellar complex (M_C_), mass fraction of the detergent (δ_D_), buoyancy mass (Mb) and expected S value can be calculated (Table 1). A lower boundary for S for monomer, dimer and trimer can be calculated considering the highest aggregation number and detergent specific volume: 0.13 S, 0.43 S and 0.99 S, respectively (last column in Table 1).

**Table 1:** Prediction of range of S values for p7b monomers, dimers and tetramers

	M_C_ (Da)	δ_D_	ν_C_ (mL/g)	MW Mb (Da)	Diameter (nm)	S (s)	S-range
Monomer	5,180 + 30,179 = 35,359	5.826	0.936	1095	4.7	0.37	**0.13–0.37**
Dimer	10,360 + 30,179 = 40,539	2.913	0.915	2136	4.9	0.69	**0.43–0.70**
Tetramer	20,720 + 30,179 = 50,899	1.456	0.886	4210	5.24	1.29	**0.99–1.29**

Prediction of S value range for the p7b oligomers. The values shown in the first six columns were obtained assuming lowest ν_D_ and aggregation number for C14SB micelles. The last column (bold) is a range of S values after considering the largest estimates of ν_D_ and aggregation number. MW of the complex (M_C_) was calculated by adding the mass of protein and lipid fraction, the mass fraction of the detergent (δ_D_) is the ratio between the mass of the detergent and that of the protein components; ν_C_ is the specific volume of the complex

AUC sedimentation equilibrium (AUC-SE) experiments were performed for 7b and 7b-TM samples in the same instrument, rotor and buffer conditions as the AUC-SV samples. For each sample, three concentrations were prepared (30, 55, and 100 µM) and centrifuged at four speeds (23,000, 28,000, 34,500, and 42,000 rpm) in 6-sector epon centerpiece AUC cells with quartz windows. Absorbance at 280 nm was measured after 24 h equilibration at each speed (confirmation of equilibrum profile was obtained after performing scans at 30 min intervals). Once obtained, the sedimentation profiles were tested with various self-association models (SEDPHAT) and plotted in GUSSI [31, 32].

The species population plot was drawn in mole fraction scale by calculating the mole fraction scale association constant K_X_ as described [33], using the expression: ${K}_{X}={K}_{A,app}\times [Det]$ where K_A,app_ is the fitted association constant in bulk molar scale, and [Det] is the concentration of micellar detergent in solution. For the monomer–dimer–tetramer equilibrium, the mole fraction of each species in the detergent phase: X_4_, X_2_, and X_1_ (tetramer, dimer, and monomer, respectively) was calculated by solving the expression below for X_1_ using the Newton–Raphson method:

$$\begin{gathered} X_{4} = \left( {K_{X,24} } \right)\left( {K_{X,12} } \right)^{2} \left( {X_{1} } \right)^{4} { } \hfill \\ X_{2} = \left( {K_{X,12} } \right)\left( {X_{1} } \right)^{2} \hfill \\ 4X_{4} + 2X_{2} + X_{1} - X_{t} = 0 \hfill \\ \end{gathered}$$

where K_X,24_ and K_X,12_ are the mole fraction scale association constants for the dimer-tetramer and monomer–dimer equilibrium, respectively, and X_t_ is the total protein mole fraction in the detergent phase. For the dimer-tetramer equilibrium, the mole fractions were similarly calculated by solving the following expression for X_2_:

$$\begin{gathered} X_{4} = \left( {K_{X,24} } \right)\left( {X_{2} } \right)^{2} \hfill \\ 2X_{4} + X_{2} - X_{t} = 0 \hfill \\ \end{gathered}$$

### Tetrameric SARS-2 p7b models in a lipid bilayer

The dimeric model of SARS-2 p7b was build using AlphaFold2 [34] server (https://colab.research.google.com/github/sokrypton/ColabFold/blob/main/AlphaFold2.ipynb), assuming α-helical struture and parallel alignment of the monomers. The distance between the two sulphur atoms of two TM cysteine residues (Cys12) was set to be close enough to form a disulphide bond. To build the initial structures of the tetramer, two possibilities were considered to orient the two homo-dimers, resulting in two different tetrameric models. The two dimers were separated by 0.85 nm to avoid clashes and placed inside a 1-palmitoyl-2-oleoyl-sn-glycero-3-phosphocholine (POPC) lipid bilayer. Lipid molecules that formed close contacts with the protein tetramer were removed. Protein parameters were based on the AMBER99SB-ILDN force field [35]. The lipid force field used is the slipid, an all-atomistic force field for biological membranes [36, 37]. The system was solvated with TIP3P [38] water molecules and counterions were added to neutralize the system. Molecular dynamics (MD) simulations were performed using GROMACS [39] 5.1.2 software. The LINCS [40] algorithm was used to constrain bonds between heavy atoms and hydrogen to enable a timestep of 2 fs. A 1.2 nm cutoff was used for Van der Waals interaction and short-range electrostatic interaction calculations, and the Particle Mesh Ewald method was implemented for long range electrostatic calculations. The simulation temperature was maintained at 300 K using a V-rescale thermostat [41] and 1 bar pressure using Parrinello-Rahman [42] barostat. Simulations of 100 ns were performed for both tetramers in the presence of the POPC lipid bilayer.

### Electrophysiology in lipid bilayers

Planar bilayers were formed by apposition of two monolayers prepared from a 5 mg/mL solution of pure 1,2-diphytanoyl-sn-glycero-3-phosphocholine (DPhPC) (Avanti polar lipids, Inc., Alabaster, AL) in pentane. Lipids were added to a ~ 100 μm diameter orifice in the 15 μm thick Teflon partition that separated two identical chambers [43, 44]. The orifice was pretreated with a 3% solution of hexadecane in pentane. Aqueous solutions consisted of 1 M KCl buffered with 5 mM HEPES at pH = 6. All measurements were performed at room temperature (23 ± 1 °C). Current events were observed after adding 0.5–1 μL of a 2.5 mg/mL solution of SARS-2 p7b in acetonitrile: H_2_O (1:1 v/v) (ACN 50%) to one side of the chamber (*cis* side). Additions were performed close to the orifice and then membrane was reformed to promote protein incorporation into the lipid bilayer. Successive additions of protein promoted always the same kind of current events. An electric potential was applied using Ag/AgCl electrodes in 2 M KCl with 1.5% agarose bridges assembled within standard 250 μL pipette tips. The potential was defined as positive when it was higher on the side of protein addition (*cis* side), whereas the *trans* side was set to ground. An Axopatch 200B amplifier (Molecular Devices, Sunnyvale, CA) in the voltage-clamp mode was used to measure the current and the applied potential. Data were filtered by an integrated low pass 8-pole Bessel filter at 10 kHz, digitized at a sampling frequency of 50 kHz with a Digidata 1440A (Molecular Devices, Sunnyvale, CA), and analyzed using pClamp 10.7 software (Molecular Devices, Sunnyvale, CA). The chamber and the head stage were isolated from external noise sources with a double metal screen (Amuneal Manufacturing Corp., Philadelphia, PA).

## Results

### SARS-2 p7b is mostly α-helical in hydrated ERGIC-like lipid bilayers

**Fig. 2:**
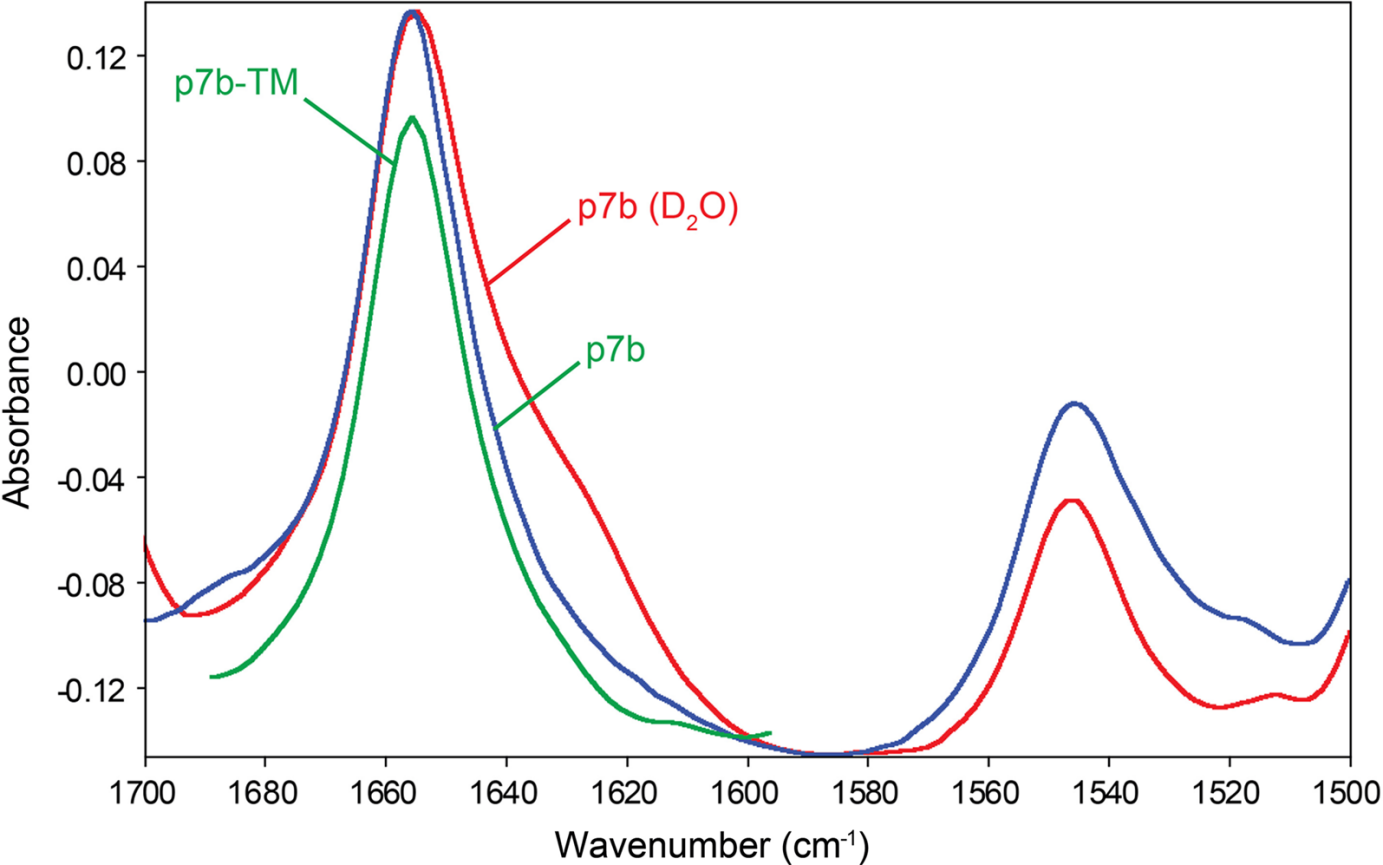
ATR-FTIR absorbance spectra of SARS-2 p7b reconstituted in ERGIC/Golgi-like lipid bilayers. ATR-FTIR spectra of p7b reconstituted in ERGIC lipid bilayers hydrated with H_2_O (blue) and D_2_O (red). For comparison, the 100% α-helical spectrum of p7b-TM is also shown (green)

When bulk water was removed, the infrared spectrum of p7b reconstituted in ERGIC-like lipid bilayers showed a highly symmetrical and narrow amide I band centered at 1656 cm^−1^, comparable to the spectrum of p7b-TM (Fig. 2). This corresponds to a completely α-helical conformation. However, hydration of the membranes with D_2_O produced a spectrum with a shoulder around 1,630 cm^−1^, suggesting a propensity for β-structure formation [45], possibly localized at the C-terminal extramembrane domain. Hydrogen–deuterium (H/D) exchange reduces the area of amide I, while keeping amide I constant. From the ratio between these two areas before and after D_2_O addition, we calculated approximately 23 amino acids resistant to H/D exchange, consistent with a predicted single TM domain (see Fig. 1). Similar results were obtained with SARS p7b and are not shown.

### Oligomerization of SARS-2 p7b in SDS gels

**Fig. 3:**
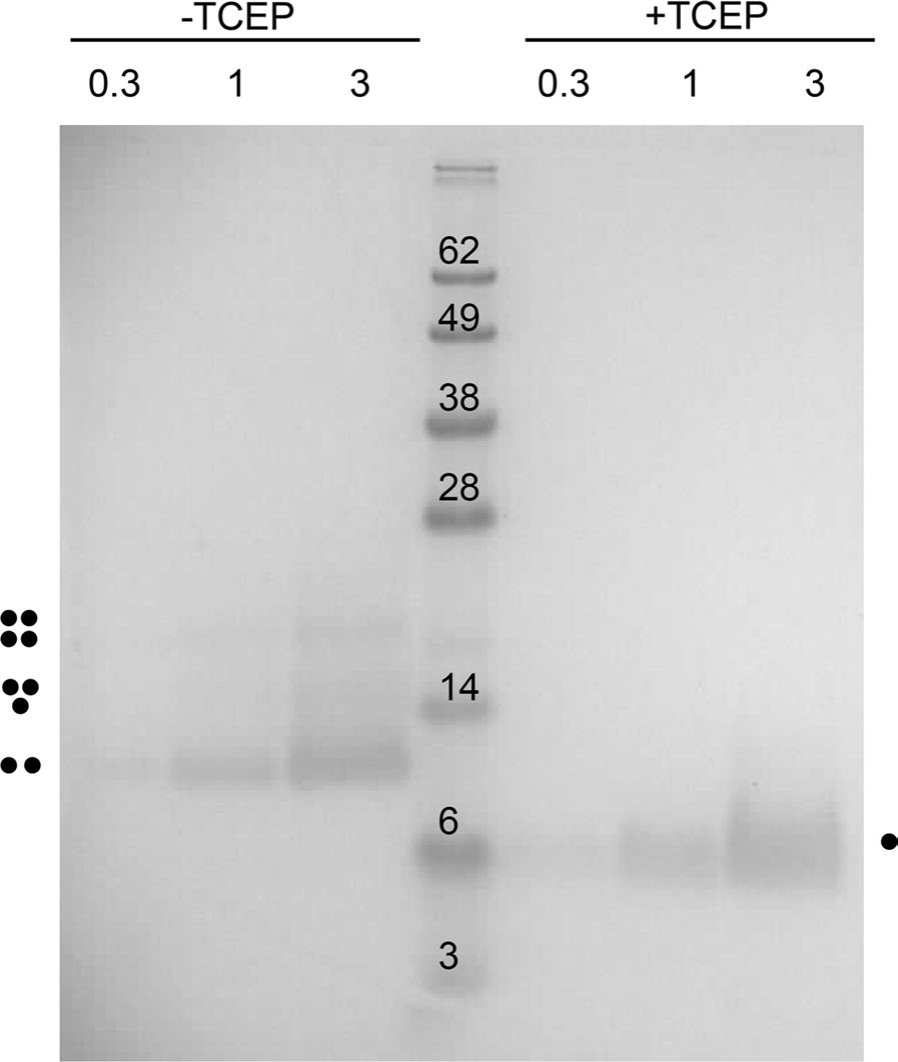
p7b forms disulfide-mediated oligomers. SDS-PAGE of SARS-2 p7b without TCEP (lanes 1–3) or with TCEP (lanes 5–7). The numbers indicate the µg used. The estimated oligomeric state is indicated by dots

Since p7b has two cysteine residues, one in the TMD and one in the extramembrane domain (Fig. 1), we tested if the pattern of migration in SDS was affected by reductants. Under non-reducing conditions (− TCEP), multiple 7b oligomers, from dimers to tetramers, were observed (Fig. 3, left). Under reducing conditions (+ TCEP), only monomers were detected (Fig. 3, right). A similar pattern was observed for SARS 7b, where higher oligomers disappeared in the presence of DTT (Additional file 1: Fig. S1). The TMD alone, which has only one cysteine residue, formed only monomers and dimers under the same conditions (Additional file 1: Fig. S1). Thus, this confirms the presence of disulfide bonds in p7b, which may have an effect on oligomerization.

### Sedimentation equilibrium of p7b in detergent micelles

**Fig. 4:**
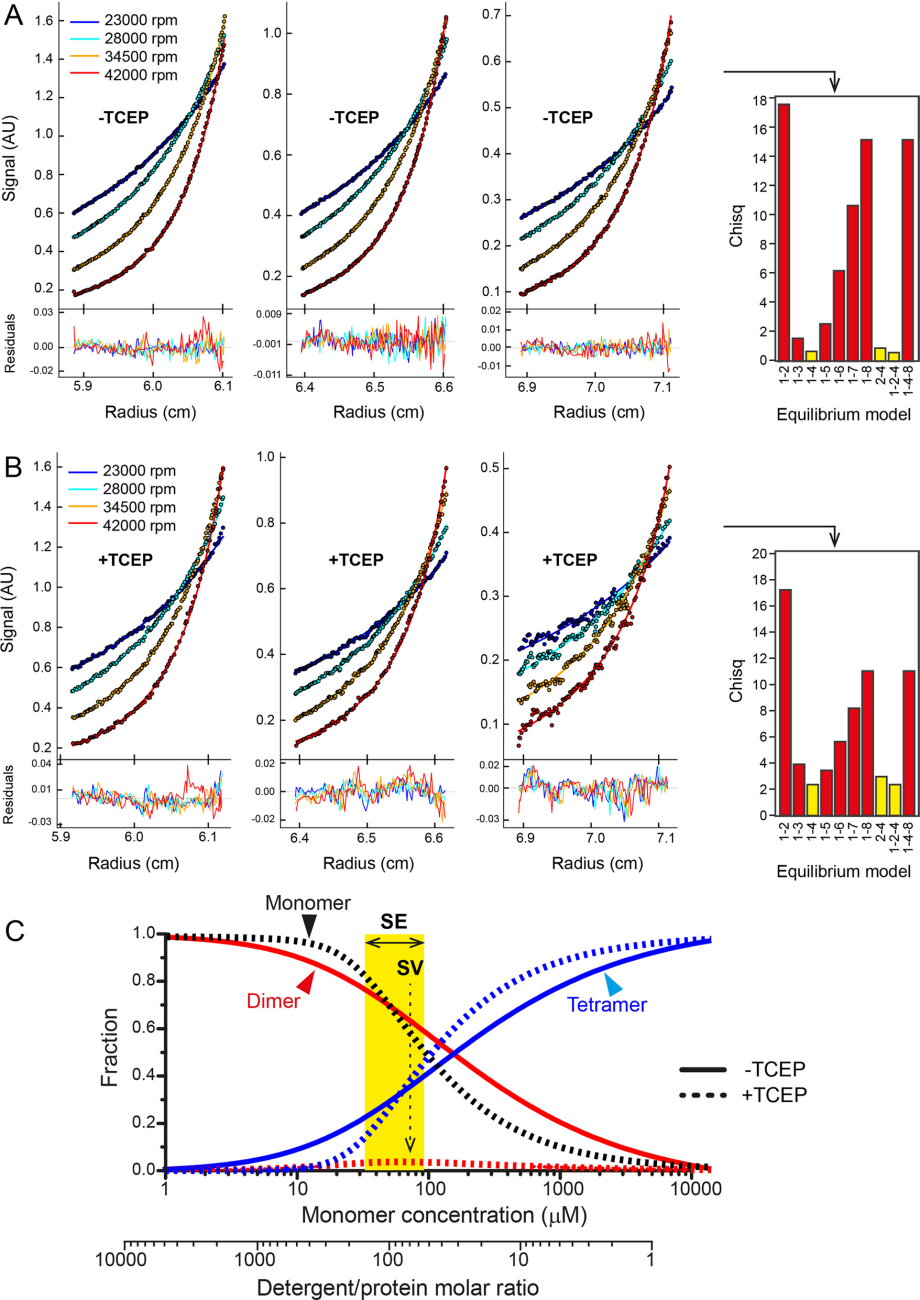
Sedimentation equilibrium profile of SARS-2 p7b in C14SB detergent. **A** Multi-speed radial distribution profile of p7b in C14SB detergent (circles). Best-fit self-association models are overlaid as solid lines in the upper panels and the fitting residuals are shown in the lower panels. The global reduced chi-square of each fitted models are shown on the bar graph on the right side, where numbers in the x axes indicate the model fitted in each case, and the best models are highlighted in yellow; **B** same as A for p7b in the presence of 2 mM TCEP; **C** monomer population distribution of p7b in oligomeric species indicated in the absence (solid line) and presence of TCEP (dotted line). The x axes indicate monomer concentration and detergent/protein molar ratio. The conditions used in SE (interval of conditions) are indicated

The oligomerization behavior of p7b was further examined by using sedimentation equilibrium (SE) where radial distribution profiles were fitted to various self-association models. In this technique, the detergent component of the sample is density-matched using D_2_O, and behavior is dependent is dependent exclusively on the molecular weight of the protein complex, not its shape. Sedimentation profiles (Fig. 4A) were fitted with several models. After an exhaustive search, the best fit was obtained with a monomer–dimer–tetramer (1–2–4) equilibrium, but also monomer-tetramer (1–4) and dimer–tetramer (2–4). Since monomers were only observed in presence of TCEP in SDS (Fig. 3), we propose that the 2–4 model as the most likely. In presence of TCEP (Fig. 4B), a similar result was observed. Since monomers are clearly observed in SDS, we chose the 1–2–4 model in this case. The distribution of these species depending on the protein-to-detergent ratio is shown in Fig. 4C, calculated using the protocol described previously [46, 47]. This data indicates that tetramer formation does not require the presence of disulfide-linked dimers, although whether tetramers with or without disulfide bonds are identical is not known (Table 2).

**Table 2:** Affinity constants in the equilibrium monomer–dimer-tetramer

Affinity constants	− TCEP	+ TCEP
K_a_ 1–2 (M^−1^)	3.867	2.791
K_a_ 2–4 (M^−1^)	5.034	6.697

Decimal logarithm of the association constants for the 1–2–4 model: monomer–dimer (1–2) and dimer–tetramer (2–4) equilibrium for SARS-2 p7b

**Fig. 5:**
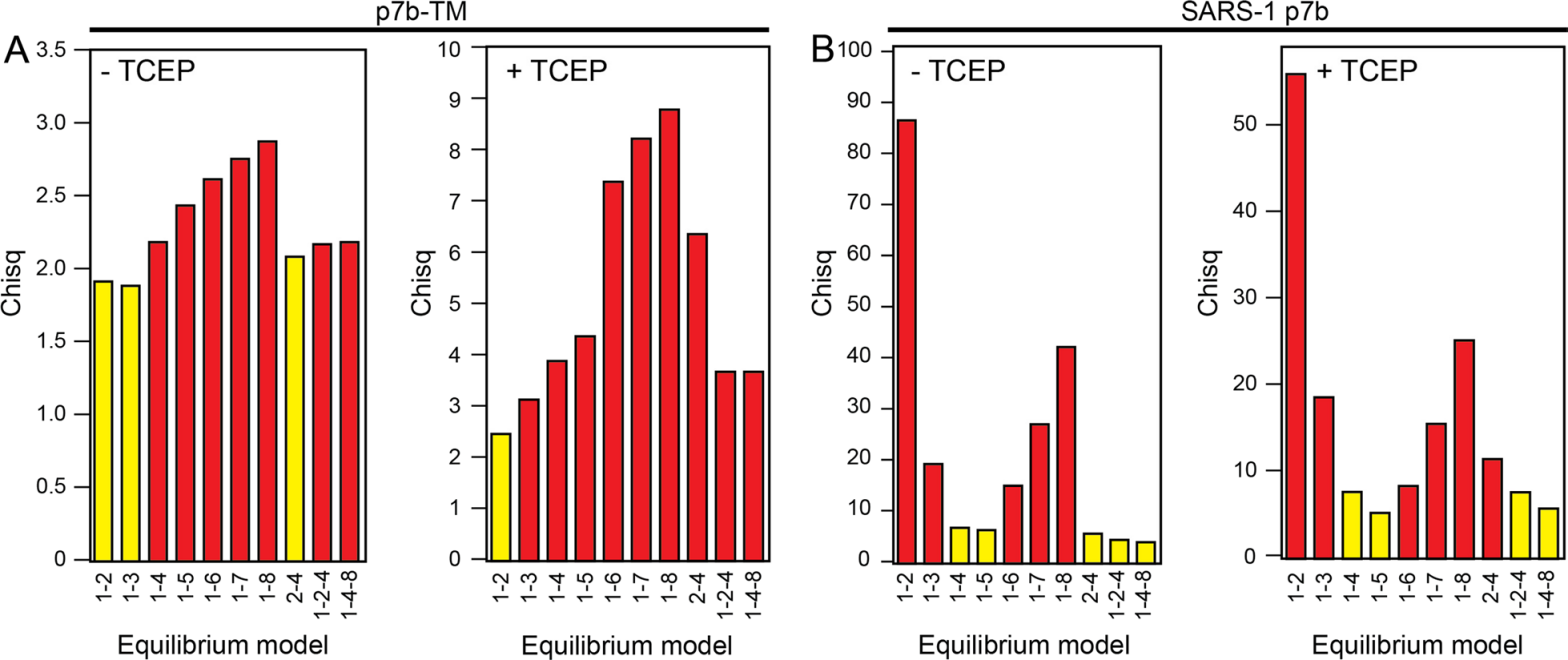
Oligomerization models for p7b-TM and SARS p7b in C14SB detergent. **A** From the multi-speed radial distribution profiles of p7b-TM (Additional file 1: Fig. S2A), best-fit self-association models were tested and the global reduced chi-square of each fitted model is shown in absence (-TCEP) and presence (+ TCEP) of 2 mM TCEP; **B** same for SARS p7b (Additional file 1: Fig. S2B). The numbers in the x axes indicate the model fitted in each case and the best models are highlighted in yellow

For 7b-TM, the best fits were 1–2, 1–3 and 2–4, but in presence of TCEP, model 1–2 was better than the other two, possibly due to the higher availability of monomer in this sample (Fig. 5A). The latter suggests that the TM domain alone can drive dimerization even in the absence of disulfide bonds, but tetramerization in p7b-TM (if any) probably requires formation of disulfide-linked dimers via Cys12. The differences between the two cases may be due to an increase in the dimer concentration when disulfide bonds are formed, or to a more favorable interaction mode between the helices in the dimer. Using the full length SARS 7b, tetramer formation does not require formation of disulfide bonds (Fig. 5B) and comparison of SARS p7b TM and p7b in presence of TCEP suggest that monomers are more abundant in the first case. Overall, this suggests (i) a significant contribution to higher oligomers (tetramers) stability of the extramembrane domains and (ii) the helix-helix orientation in the dimer may be the same, with or without disulfide bonds.

### Sedimentation velocity of p7b in detergent micelles

**Fig. 6:**
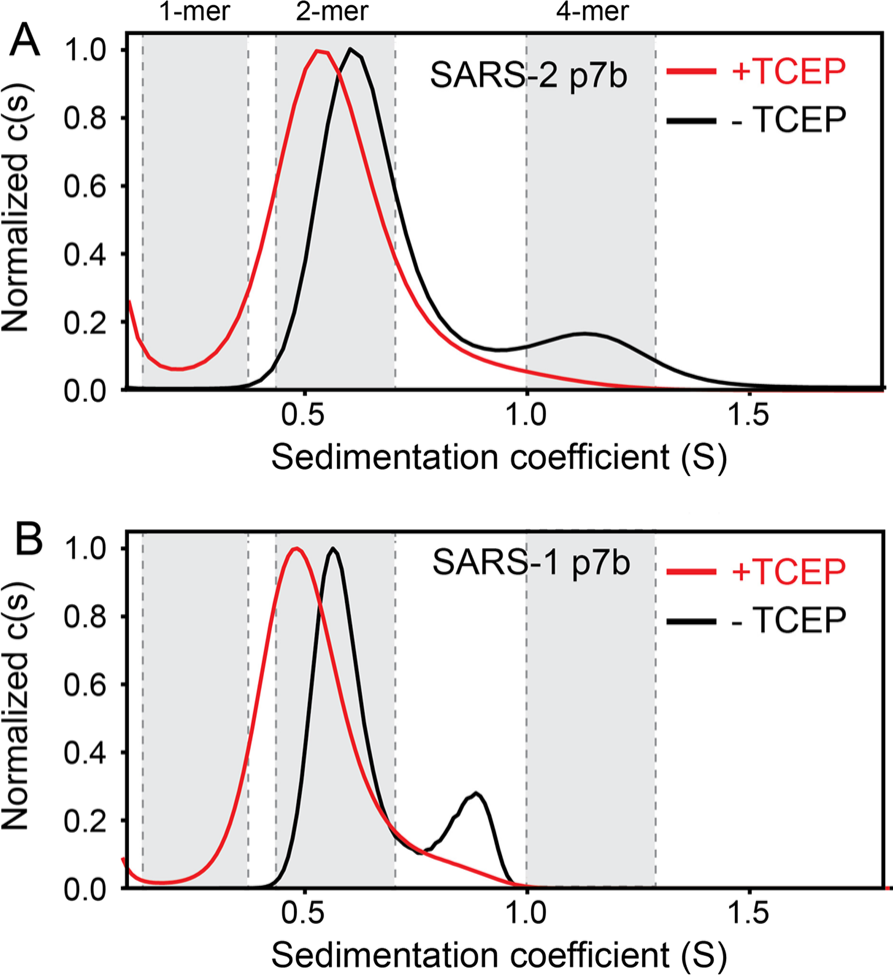
Sedimentation velocity profile of SARS-2 and SARS p7b in C14SB detergent. (A) Comparison of c(s) distribution obtained with (red) or without reductant TCEP (black). Grey rectangles indicate the range of S-values calculated for a p7b monomer, dimer and tetramer (see Methods section); (B) same for SARS p7b

Although SE is the AUC gold standard to determine molecular weights of membrane proteins, the ambiguity in the models proposed suggested the use of SV to complement the SE data. In the absence of reductant TCEP, SARS-2 p7b produced two bands in the c(s) distribution profile (Fig. 6A), one at ~ 0.55 S, consistent with the size of a dimer (2-mer) and the other at 1.1 S consistent with a tetrameric form (4-mer). The latter is in principle consistent with the SE results (Fig. 4). In presence of TCEP, the proposed tetramer (1.1 S) disappeared, and the lower-S species shifted to ~ 0.5 S, still consistent with a dimer. However, we note that a single band in the c(s) plot can also indicate a rapid equilibrium beween smaller and larger species [48], therefore a rapid equilibrium between monomers, dimers and tetramers is still compatible with this data. In any case, this indicates that oligomerization does not strictly require disulfide bond formation. The fact that this band is slightly shifted relative to the no-TCEP condition, further suggests that it may represent, not a dimer, but a fast equilibrium between the monomer, dimer and tetramer [48]. Thus, two possible models emerge where a tetramer is formed by two dimers: (i) in one, disulfide bonds elicit dimer formation, prior to tetramer formation; (ii) in the other, interaction between the dimers is non-covalent, whereas disulfide bonds join two dimers. A similar pattern of dimers and tetramers were also observed in the case of SARS p7b (Fig. 6B). Here, the result was slightly different because the larger S band was located in the ‘trimer’ region. As discussed above, this is again consistent with an intermediate species resulting from the fast exchange between dimers and tetramers, reflecting the extramembrane domain differences between these two sequences and further supporting that the extramembrane domain is involved in the formation of these oligomers.

### Model building

**Fig. 7:**
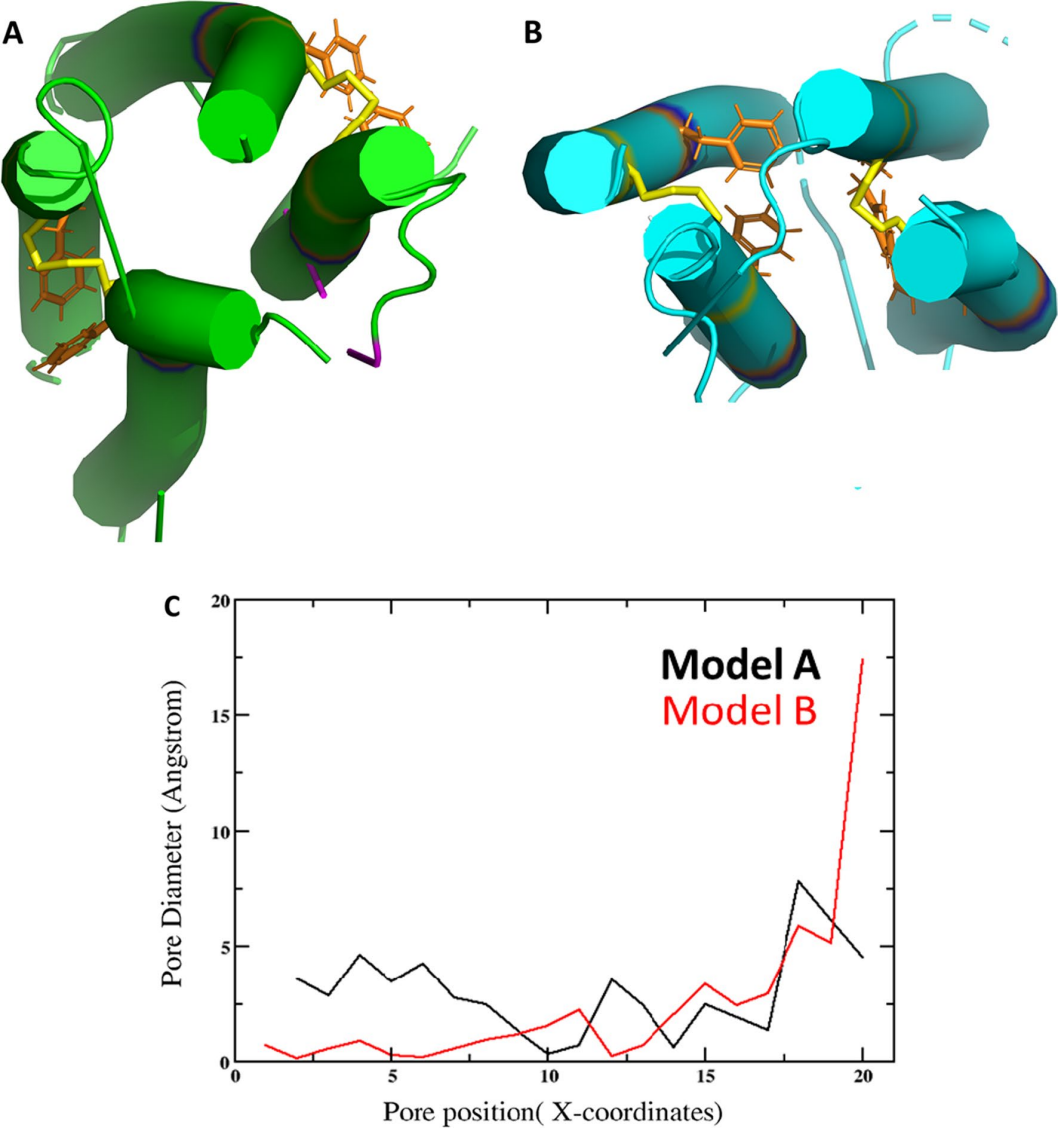
Models of 7b-TM homotetramer.** A**–**B** Two tetrameric models obtained by interaction of two disulfide-linked dimers (yellow). The dimers were obtained with Alpha-Fold, whereas the tetramer was embedded in a POPC membrane;** C** the pore diameter reaches zero in several parts along the channel for both models (lower panel)

We generated dimers linked by Cys 12 in AlphaFold and neither of these tetrameric models (Fig. 7) produced a structure compatible with a channel (https://mole.upol.cz/).

### Lipid bilayer permeabilization

**Fig. 8:**
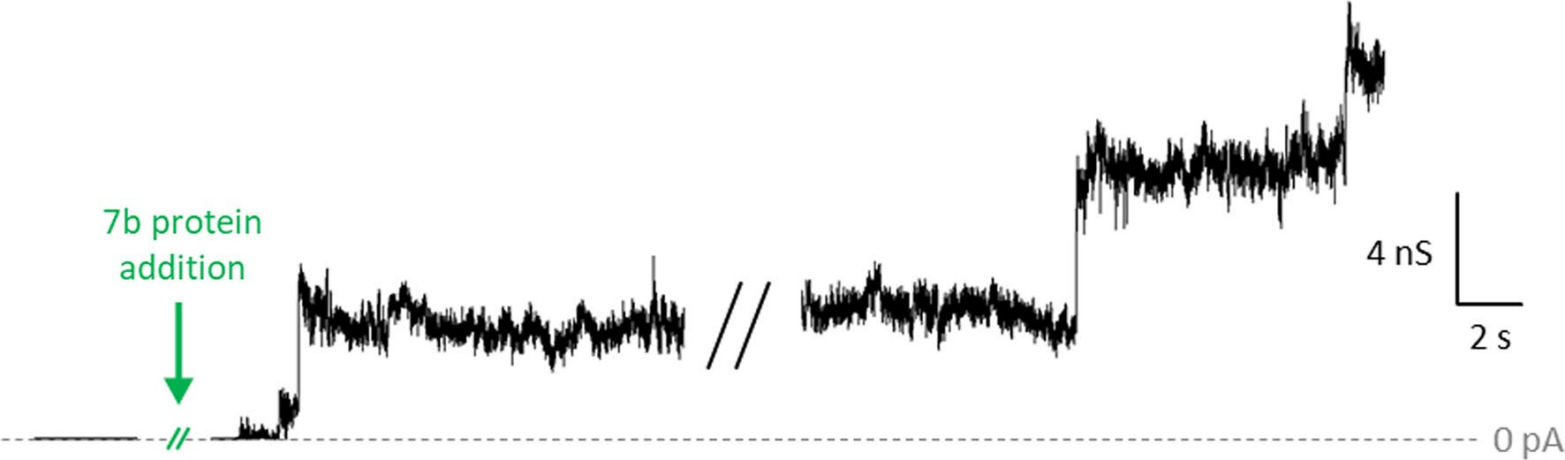
Occasional lipid membrane permeabilization by p7b. Representative current recordings with applied voltage + 100 mV after addition of p7b to DPhPC planar lipid membranes. Recordings were digitally filtered with a low-pass 8-pole Bessel filter with cut-off frequency of 500 Hz

Planar membrane electrophysiology was used to test full-length SARS-2 p7b ability to form channels in lipid bilayers. Addition of the protein diluted in ACN 50% induced only occasional large unstable currents lasting over minutes and presenting stepwise current transitions (Fig. 8). In general, these currents did not produce membrane rupture. p7b-induced current activity was observed at applied voltages ranging from ± 10 to ± 100 mV. Control experiments with ACN 50% alone did not produce any effect on the membrane. The conductance (G = I/V) measured during p7b-induced membrane permeabilization events was of several nanoSiemens, with typical conductance steps of 1–5 nS (Fig. 8). Such high conductances are comparable to those measured in wide porins with diameters of 1–2 nm [49, 50]. However, the instability observed in p7b-induced currents does not resemble that of porins or other channel forming proteins such as SARS-CoV-2 E protein [51], which show quieter currents. An explanation for the appearance of such large transient current levels –maybe related to high protein/lipid ratio, promoting a detergent-like action [52]– is out of the scope of the present work. Thus, the observed p7b-induced bilayer permeabilization evidences that the protein interacts with lipid membranes, but it is hardly compatible with the existence of bona fide ion channels based on a tetrameric assembly with several hydrophobic residues lining the narrow pore.

## Discussion

The precise role during infection of the group-specific accessory proteins encoded by SARS-CoVs remains incompletely understood. However, since SARS-CoV genomes encode the largest number of accessory proteins among coronaviruses, it is tempting to speculate that they have some important role in the clinical manifestation of infection. SARS-CoV 3a, 6, 7a, and 7b have transmembrane domains [18, 26, 53, 54]. 7a has a high structural similarity to the Ig-like superfamily proteins, although no significant sequence homology [55, 56], whereas 3a forms tetrameric ion channels [57] with a structure in lipid nanodiscs recently solved using cryo-EM [58]. In the present study we have investigated the behavior of p7b in both lipid and detergent environments to assess whether it forms oligomers with ion channel properties. Overall, the conclusion from our data is that p7b is predominantly α-helical, although the extramembrane domain may form some β-strands. This latter domain likely contributes to the stability of the oligomers.

Although oligomers are observed in SDS, this strong detergent and not a suitable environment to study the assembly of α-helices. However, it is clear that no monomers are present in the absence of reductant. This eliminates models involving monomers when fitting SE sedimentation profiles that use the milder detergent C14SB. Therefore, we suggest that the most likely model is an equilibrium between dimers and tetramers. When a reductant is present, only monomers were observed in SDS, and clear changes were also observed in SV experiments. This confirms that disulfide bonds are present in the sample, and that disruption of these bonds affects oligomerization, but does not prevent tetramer formation, since the best model in AUC SE is 1–2–4. Here, the model may be complicated by having two types of dimers (linked or not linked by disulfide bonds), small contribution of trimers, or larger oligomers, contribution of TCEP itself, and so on. However, we feel it is out of the scope of this paper to characterize further such complex behavior.

Although the involvement, but not the requirement, of disulfide bonds in oligomerization is reminiscent of that of Influenza virus A M2 proton channel, no pore pathway was detected in any of the proposed tetrameric models. In addition, this is supported experimentally since the electrical activity detected in standard bilayers was not consistent with a very narrow tetrameric pore, suggesting that p7b does not form bona fide ion channels. Overall, the presence of DTT-resistant dimer in gel electrophoresis and in SE experiments in presence of reductant suggests that dimerization does not require disulfide bond formation, although the latter may stabilize it. In a similar system, the cysteine residue in the ζζ transmembrane domain was suggested to stabilize the dimeric form, only after formation of proper interface [59]. Overall, the role played by the p7b in the viral life cycle, and during infection of SARS-CoV is still unclear, but we provide a first glimpse of its oligomerizing behavior.

## Supplementary Information

**Additional file 1: Fig. S1.** SDS-PAGE electrophoresis of SARS-1 p7b-TM and p7b, with or without DTT. The peptides were subjected to SDS-PAGE using 16.5 % precast tricine gel (Bio-Rad), with or without 1,4-dithiothreitol (DTT). SDS sample buffer was added to the lyophilized peptide to a final concentration of 2 μg/uL. The sample was mixed with sample buffer for 1 min followed by heating at 95 °C for 5 min before loading to the gel. The gel was run at constant voltage of 80 V for 3 h at room temperature. The molecular mass markers were obtained from Invitrogen (Thermo Fisher Scientific). The gel was stained with Coomassie blue. Left lanes are p7b-TM; lane 3 is molecular mass marker; lanes 4-5 are p7b. **Fig. S2.** Sedimentation equilibrium profile of SARS1 7b-TM and 7b in C14SB detergent. **A** Radial distribution profile of 7b-TM in C14SB at 28000 rpm (red circles), 34500 rpm (green circles), and 42000 rpm (blue circles). The presence of TCEP is indicated in the respective panels. Best-fit self-association models are overlaid as solid lines in the upper panels and the fitting residuals are shown in the lower panels. Best-fit model for 7b-TM without TCEP was a dimer-tetramer whereas with TCEP it was a monomer-dimer; **B** The same as A for SARS1 7b, where the best-fit model was a monomer-dimer-tetramer with or without TCEP. We note that sometimes the fitting residuals are not randomly distributed even in the best-fit model. This indicates there could still be a small amount of other species unaccounted for, possibly intermediate species (e.g., trimer) or higher order oligomers (e.g., octamer), which are too complex to model alongside the dimer and tetramer

## Data Availability

The data will be shared on a reasonable request to the corresponding author.
