# Supplementary material for: SARS-CoV-2 accessory protein 7b forms homotetramers in detergent
Source: Virol J. 2022 Nov 21;19:193. doi: 10.1186/s12985-022-01920-0 (PMC9680129; doi:10.1186/s12985-022-01920-0)
Supplement: Supplementary file 1 — Additional file 1: Fig. S1. SDS-PAGE electrophoresis of SARS-1 p7b-TM and p7b, with or without DTT. The peptides were subjected to SDS-PAGE using 16.5 % precast tricine gel (Bio-Rad), with or without 1,4-dithiothreitol (DTT). SDS sample buffer was added to the lyophilized peptide to a final concentration of 2 μg/uL. The sample was mixed with sample buffer for 1 min followed by heating at 95 °C for 5 min before loading to the gel. The gel was run at constant voltage of 80 V for 3 h at room temperature. The molecular mass markers were obtained from Invitrogen (Thermo Fisher Scientific). The gel was stained with Coomassie blue. Left lanes are p7b-TM; lane 3 is molecular mass marker; lanes 4-5 are p7b. Fig. S2. Sedimentation equilibrium profile of SARS1 7b-TM and 7b in C14SB detergent. A Radial distribution profile of 7b-TM in C14SB at 28000 rpm (red circles), 34500 rpm (green circles), and 42000 rpm (blue circles). The presence of TCEP is indicated in the respective panels. Best-fit self-association models are overlaid as solid lines in the upper panels and the fitting residuals are shown in the lower panels. Best-fit model for 7b-TM without TCEP was a dimer-tetramer whereas with TCEP it was a monomer-dimer; B The same as A for SARS1 7b, where the best-fit model was a monomer-dimer-tetramer with or without TCEP. We note that sometimes the fitting residuals are not randomly distributed even in the best-fit model. This indicates there could still be a small amount of other species unaccounted for, possibly intermediate species (e.g., trimer) or higher order oligomers (e.g., octamer), which are too complex to model alongside the dimer and tetramer [file 12985_2022_1920_MOESM1_ESM.docx]

**SUPPLEMENTARY INFORMATION**


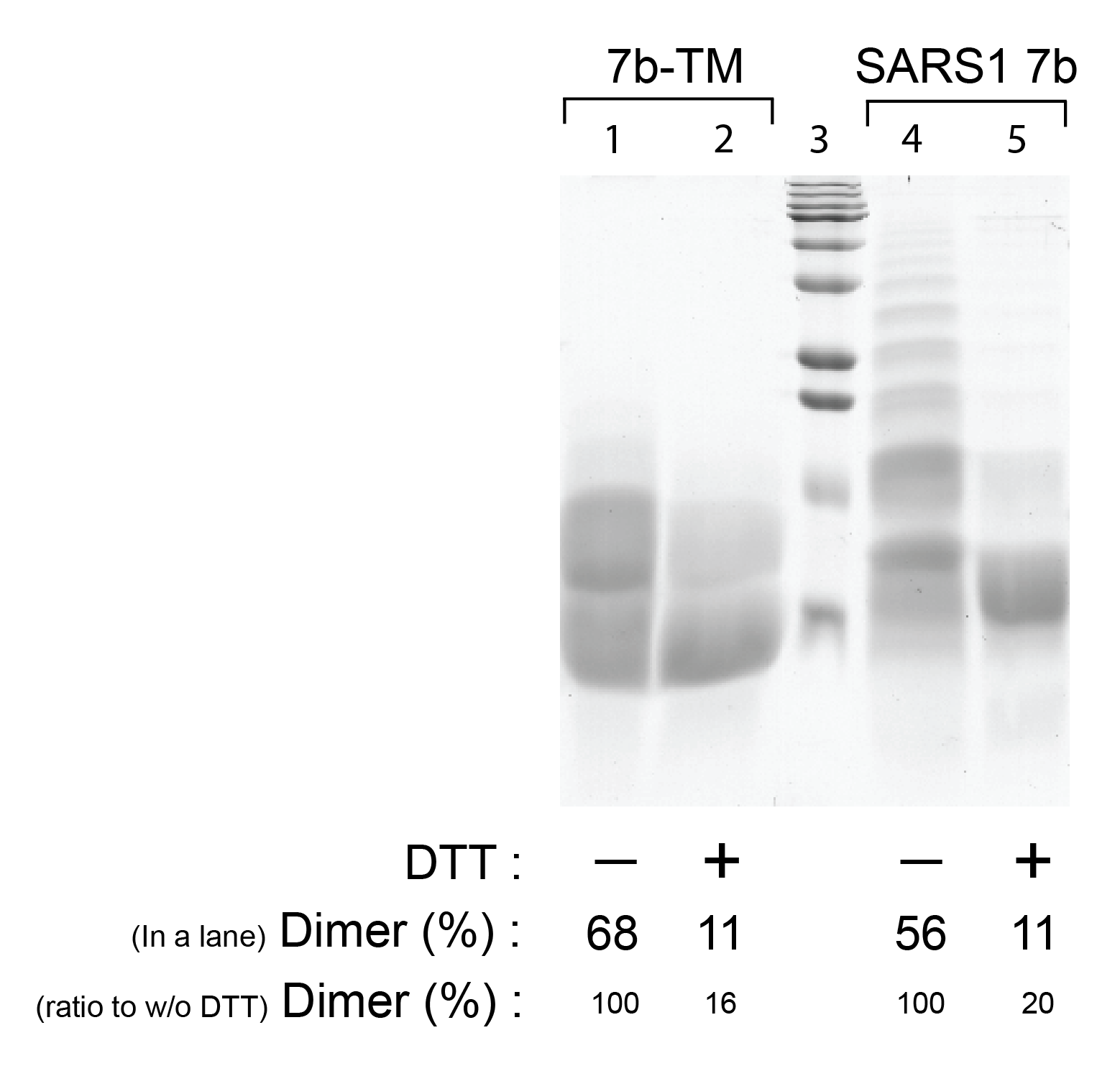


**Figure S1.** **SDS-PAGE electrophoresis of SARS-1 p7b-TM and p7b, with or without DTT.** The peptides was subjected to SDS-PAGE using 16.5 % precast tricine gel (Bio-Rad), with or without 1,4-dithiothreitol (DTT). SDS sample buffer was added to the lyophilized peptide to a final concentration of 2 μg/uL. The sample was mixed with sample buffer for 1 min followed by heating at 95°C for 5 min before loading to the gel. The gel was run at constant voltage of 80 V for 3h at room temperature. The molecular mass markers were obtained from Invitrogen (Thermo Fisher Scientific). The gel was stained with Coomassie blue. Left lanes are p7b-TM; lane 3 is molecular mass marker; lanes 4-5 are p7b.

**
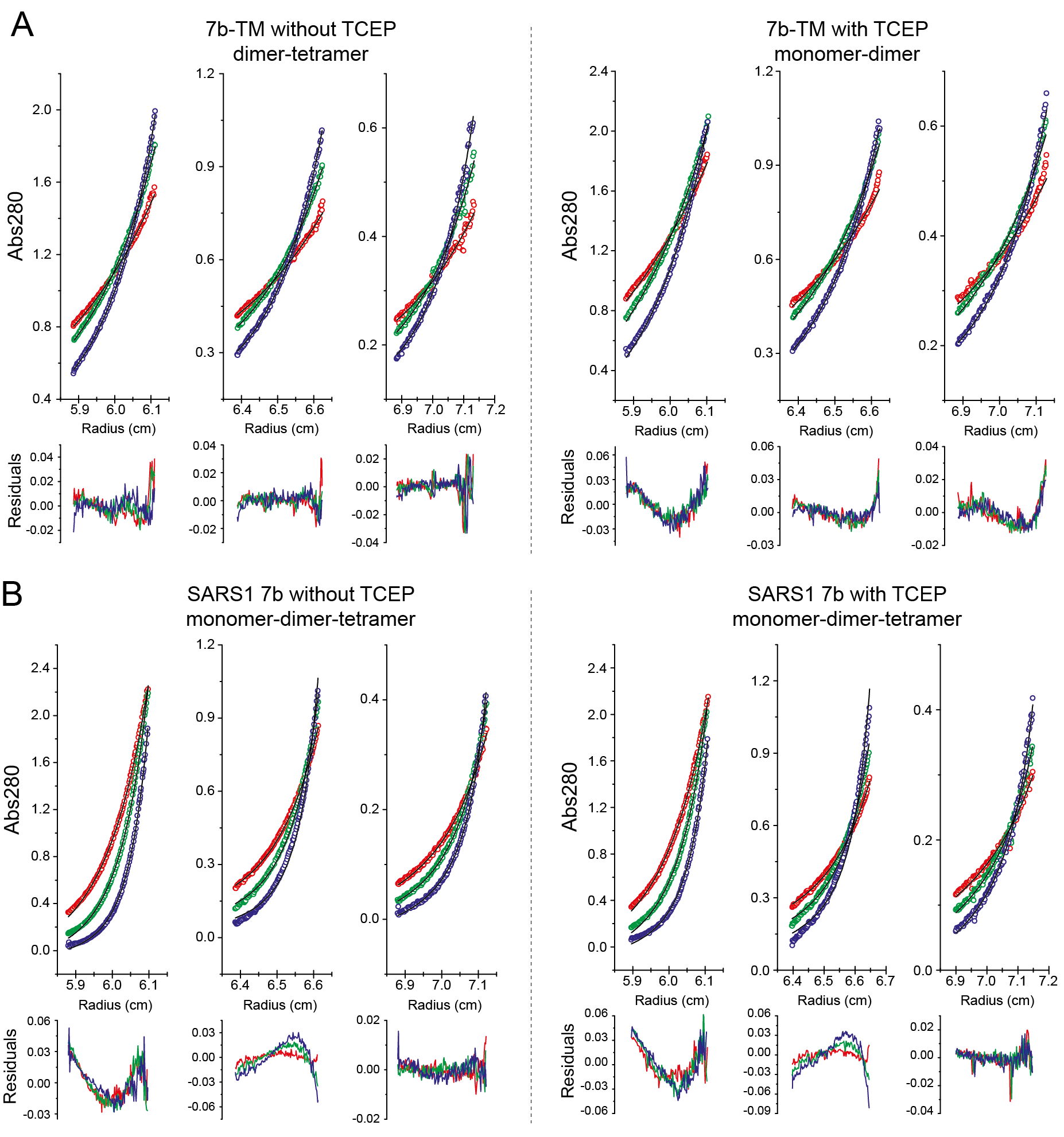
**

**Figure S2. Sedimentation equilibrium profile of SARS1 7b-TM and 7b in C14SB detergent.** *(A)* Radial distribution profile of 7b-TM in C14SB at 28000 rpm (red circles), 34500 rpm (green circles), and 42000 rpm (blue circles). The presence of TCEP is indicated in the respective panels. Best-fit self-association models are overlaid as solid lines in the upper panels and the fitting residuals are shown in the lower panels. Best-fit model for 7b-TM without TCEP was a dimer-tetramer whereas with TCEP it was a monomer-dimer; *(B)* The same as A for SARS1 7b, where the best-fit model was a monomer-dimer-tetramer with or without TCEP. We note that sometimes the fitting residuals are not randomly distributed even in the best-fit model . This indicates there could still be a small amount of other species unaccounted for, possibly intermediate species (e.g., trimer) or higher order oligomers (e.g., octamer), which are too complex to model alongside the dimer and tetramer.
